# Genotype Triad for *HOTAIR* rs10783618, *LINC-ROR* rs1942347, and *MALAT1* rs3200401 as Molecular Markers in Systemic Lupus Erythematous

**DOI:** 10.3390/diagnostics12051197

**Published:** 2022-05-11

**Authors:** Nesreen M. Ismail, Eman A. Toraih, Amany I. Almars, Essam Al Ageeli, Manal S. Fawzy, Shymaa Ahmed Maher

**Affiliations:** 1 Department of Rheumatology and Rehabilitation, Faculty of Medicine, Suez Canal University, Ismailia 41522, Egypt; nisreenmohamed@med.suez.edu.eg; 2 Division of Endocrine and Oncologic Surgery, Department of Surgery, Tulane University School of Medicine, New Orleans, LA 70112, USA; 3 Genetics Unit, Department of Histology and Cell Biology, Suez Canal University, Ismailia 41522, Egypt; 4 Department of Medical Laboratory Sciences, Faculty of Applied Medical Sciences, King Abdulaziz University, Jeddah 21589, Saudi Arabia; aialmars@kau.edu.sa; 5 Department of Clinical Biochemistry (Medical Genetics), Faculty of Medicine, Jazan University, Jazan 45142, Saudi Arabia; dr.ageeli@gmail.com; 6 Department of Biochemistry, Faculty of Medicine, Northern Border University, Arar 1321, Saudi Arabia; 7 Department of Medical Biochemistry and Molecular Biology, Faculty of Medicine, Suez Canal University, Ismailia 41522, Egypt; shimaa.maher@med.suez.edu.eg; 8 Center of Excellence in Molecular and Cellular Medicine (CEMCM), Faculty of Medicine, Suez Canal University, Ismailia 41522, Egypt

**Keywords:** *HOTAIR*, *LINC-ROR*, long non-coding RNAs, lupus nephritis, *MALAT1*, single nucleotide polymorphism, SLE

## Abstract

Accumulating evidence supports the implication of long non-coding RNAs (lncRNAs) in autoimmune diseases, including systemic lupus erythematosus (SLE). LncRNA variants could impact the development and/or outcome of the disease with variable diagnostic/prognostic utility in the clinic. We aimed to explore the contribution of *HOTAIR* (rs10783618), *LINC-ROR* (rs1942347), and *MALAT1* (rs3200401) variants to SLE susceptibility and/or severity in 163 SLE patients and age-/sex-matched controls using real-time TaqMan allelic discrimination PCR. *HOTAIR* rs10783618*C/C was associated with a 77% increased risk of SLE (OR = 1.77, 95%CI = 1.09–2.87, *p* = 0.020) under the recessive model. Similarly, *MALAT1* rs3200401*T/T carriers were three times more likely to develop SLE (OR = 2.89, 95%CI = 1.42–5.90) under the recessive model. While the rs3200401*T/C genotype was associated with a 49–57% decreased risk of SLE under codominant (OR = 0.51, 95%CI = 0.31–0.82, *p* < 0.001) and over-dominant (OR = 0.43, 95%CI = 0.27–0.68, *p* < 0.001) models. *LINC-ROR* rs1942347*A/A patients were more likely to have a positive family history of SLE. At the same time, *HOTAIR* rs10783618*C/C was associated with a higher frequency of arthritis (*p* = 0.001) and the presence of oral ulcers (*p* = 0.002), while patients carrying rs10783618*T/T genotype were more likely to develop hair loss (*p* < 0.001), weight loss (*p* = 0.001), and neurological symptoms (*p* = 0.003). In conclusion, the studied lncRNAs, *HOTAIR,* and *MALAT1* gene polymorphisms confer susceptibility for SLE, providing a potential theoretical basis for their clinical translation in SLE disease.

## 1. Introduction

Systemic lupus erythematosus (SLE) is a complex, chronic, potentially fatal, multisystem autoimmune disease that predominantly affects women between puberty and menopause [1]. The mortality rate in SLE patients is relatively high [2], and delay in diagnosis is associated with increased damage to vital organs [3]. Accumulating evidence indicates that the interaction of genetic/epigenetic factors with the environmental and immunological insults is required for disease development [4,5,6,7,8,9]. SLE and other similar disorders such as rheumatoid arthritis (RA), Sjogren’s syndrome (SS), and celiac disease, in which autoimmunity, inflammation, and immunosuppressive therapy use are the hallmarks of these conditions, have been associated with cancer, including non-Hodgkin lymphoma [10,11,12]. This association has been reported to have a mutual relationship, indicating a disease-specific risk profile and having genetic determinants contributing to increased disease morbidity and mortality [11,12].

“Genome-wide association studies; GWAS” and “next-generation sequencing (NGS) studies” have uncovered >100 SLE susceptibility loci and candidate genetic variants associated with SLE development [13,14]. Surprisingly, few such variants have been identified to derange the coding genes with subsequent loss/gain of function for the encoded proteins, while most variants enriched within the non-coding sequences have the potential to impact gene expression at the transcriptional post-transcriptional and/or translational levels [15,16].

Long non-coding RNAs (lncRNAs) are molecules longer than 200 nucleotides in length, with no protein-coding capacity. LncRNAs are known to regulate gene expression and to play an essential role in the regulation of many biological processes at the transcriptional and post-transcriptional levels [7]. They could interact with proteins in the cytoplasm as a guide, scaffold, or decoy molecules [17].

As lncRNA could affect T cell differentiation and function [7], and T cells play a central role in cell-mediated immune response, any abnormality in T cell function could impact SLE [18]. Recently, many studies showed that several lncRNAs and related variants could be implicated in the pathogenesis of SLE [8,9,19]. 

In this preliminary study, based on our in silico analyses and searches of previous literature for some lncRNAs-related variants that have not been extensively explored in SLA [9,20,21,22], the following LncRNA variants were selected: (1) the lncRNA HOX transcript antisense RNA (*HOTAIR*) rs10783618, and (2) the intergenic lncRNA regulator of reprogramming (*LINC-ROR*) rs1942347, which have been associated with autoimmune diseases but no or little studies have been explored with SLE [23,24,25], as well as (3) metastasis-associated lung adenocarcinoma transcript 1 (*MALAT1*) rs3200401. This lncRNA is an abundantly expressed nuclear lncRNA and has been reported to significantly affect monocyte and inflammatory cytokines levels in SLE patients [26].

Despite growing evidence worldwide emphasizing the essential roles that lncRNAs could play in autoimmune and inflammatory diseases [11], our knowledge of SLE-related lncRNAs remains limited in the Middle East. In this sense, this study aimed to explore the contribution of the lncRNA-related variants mentioned above to SLE susceptibility and/or severity in a sample of the Middle East population.

## 2. Materials and Methods

### 2.1. Study Subjects

The current study included 163 SLE patients and 163 age- and sex-matched blood donor controls. SLE patients were recruited from the “Rheumatology and Nephrology Departments, the Suez Canal University (SCU) Hospitals, Ismailia, Egypt”. Patients were diagnosed and followed by experienced Rheumatologists according to the 2019 European League Against Rheumatism (EULAR)/American College of Rheumatology (ACR) classification criteria for SLE [27]. SLE patients were clinically assessed for eligibility. Patients with concomitant chronic or autoimmune disorders were excluded (i.e., seven SLE patients: two patients with diabetes mellitus type 2, one patient with rheumatoid arthritis, one patient with bronchial asthma, three patients with hypothyroidism). History, examination, and laboratory data were collected. Disease activity was graded based on the “SLE Disease Activity Index (SLEDAI) score” [28]. Lupus nephritis was diagnosed according to the ACR criteria [29], and patients were stratified into two subgroups accordingly. The control group included 163 age- and sex-matched healthy blood donors. They were attending the blood bank of the SCU hospital in the same period with no history of chronic disorders, including autoimmune diseases. The authors followed the “Helsinki declarations” during work execution, and the study was approved by the local Institutional ethical committee (approval no. #3962). Informed written consent was obtained from enrolled study subjects prior to the research.

### 2.2. Selection of the Study Genetic Variants

The top frequent single nucleotide polymorphism for each gene in Ensembl Genome Browser (www.ensembl.org 20 August 2021) was the main selection criteria used. *HOTAIR* rs10783618 C/T was the most common SNP, with MAF accounting for 0.50. The SNP covers all transcript isoforms and thus was enrolled. For the *LINC-ROR* gene, the most frequent SNP was rs8093490 at 18:57052460 with a minor allele frequency (MAF) of 0.462; however, it represents three alternative alleles (A/G/T). Therefore, the second most frequent SNP rs1942347 (A/T) at 18:57057227 with a MAF of 0.467 was selected. Regarding the *MALAT1* gene, genetic variants were sorted by MAF, and indel mutations were filtered out. The most prevalent biallelic SNP was rs591291C/T, with a MAF of 0.498 at 11:65497011, which overlaps only 14 of the 25 *MALAT1* gene transcripts. The second common SNP, rs3132742 A/G with MAF of 0.467 located at 11:65495530, overlaps only 13 transcript isoforms. We finally selected the next common biallelic SNP rs3200401 T/C at 11:65504361 with a MAF of 0.143, which was cited 21 times and showed association with cancer and noncancer disorders. 

### 2.3. Allelic Discrimination Analysis

Whole blood samples (5 mL) were collected in EDTA vacutainers, and DNA was extracted from the buffy coat using the QIAamp DNA extraction Mini kit (Qiagen; Catalog #: 51104) according to the manufacturer’s instructions. The purity/concentration of isolated DNA was evaluated by NanoDrop ND-1000 spectrophotometer (NanoDrop Technologies, Inc., Wilmington, DE, USA). Real-Time allelic discrimination polymerase chain reaction (PCR) was carried out using TaqMan assays for the *HOTAIR* rs10783618 (C___2104248_10) which detect the C/T transition substitution in the genomic context sequence [VIC/FAM]: “TACAATTTTTTGTGTCCTCCTTATC[C/T]GGTTTGGGAGCCGCAGCACCTTATC”, the *LINC-ROR* rs1942347 (C__11450075_10) which determines the transversion A/T substitution in the sequence [VIC/FAM]: “GGTGTATACCTAGGAGCAAAGTTGC[A/T]GGGTCATATGGGAACCCTATGTTTA”, and the MALAT1 rs3200401 (C___3246069_10) which detect the T/C transition substitution in the context sequence [VIC/FAM]: “GAATGCAGTTGTCTTGACTTCAGGT[T/C]TGTCTGTTCTGTTGGCAAGTAAATG” according to the build GRCh38 as described in details in our previous publication [27]. The real-time PCR was performed blinded to the case/control status of the samples in a StepOne Real-Time PCR System (Applied Biosystems, Foster City, CA, USA). The PCR program was set at 95 °C (10 min), followed by 40 cycles of 95 °C (15 s), 60 °C (1 min), and 60 °C (30 s). Appropriate controls (no template and no enzyme) were added to each run. Ten percent of random samples were reanalyzed, which yielded a 100% concordance rate. The SDS software version 1.3.1. (Applied Biosystems, Foster City, CA, USA) was applied for genotyping data analysis.

### 2.4. Statistical Analysis

Statistical analyses were performed by GraphPad Prism v9.0 and Statistical Package for Social Science (version 27.0). Two-sided Chi-square and Student’s *t*-tests were used in the analysis. Hardy–Weinberg equilibrium (HWE) was estimated. Genotype and allele frequencies were estimated, and five genetic inheritance models were investigated as previously published [30]. SNPstats software was applied [31]. Both crude and adjusted (by age and sex) regression analyses were employed to test for disease risk. Odds ratio (OR) and 95% confidence interval (CI) were represented. *p* ≤ 0.05 was considered statistically significant. The principal component analysis was plotted using R packages. 

## 3. Results

### 3.1. Characteristics of the Study Population

The study included 163 SLE patients (147 females and 16 males) and 163 age and sex-matched controls (148 females and 15 males). Their mean age was 35.6 ± 9.6 years for patients and 35.8 ± 9.9 years for controls. Fifty-eight (35.6%) cases had a positive family history of SLE, and 112 (68.7%) patients had an early-onset disease. Around 90% and 99% of cohorts were positive for dsDNA and ANA, respectively. In the patient group, 98 (60.1%) had lupus nephritis disease. They were more likely to be females (94.9% vs. 83.1%, *p* = 0.016) and have positive dsDNA autoantibodies (98% vs. 78.5%, *p* < 0.001) compared to SLE patients without nephritis. The lupus nephritis group showed higher disease activity score (18.63 ± 10.21 vs. 12.03 ± 7.75, *p* < 0.001), serum creatinine (57.1% vs. 24.6%, *p* < 0.001), and proteinuria (78.6% vs. 23.1%, *p* < 0.001). A comparison between SLE patients with and without nephritis is demonstrated in Table 1. 

### 3.2. LncRNAs Genotype and Allelic Frequencies

**Figure 1:**
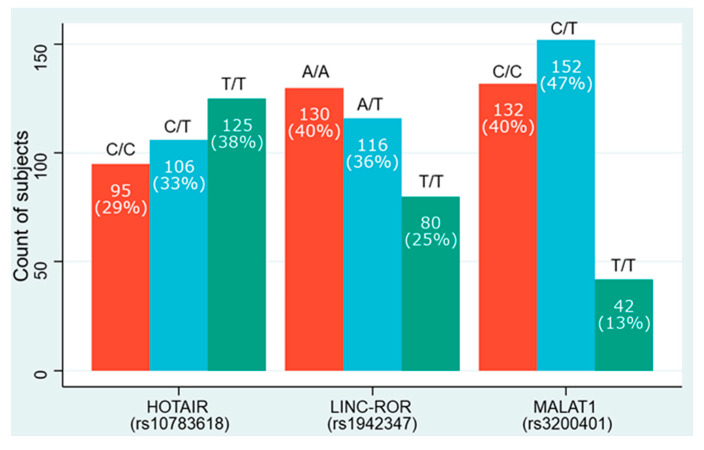
Genotype frequency of lncRNA genotypes in the study population. Data are presented as count (percentage).

Genotype frequencies were in agreement with Hardy–Weinberg equilibrium (*p* > 0.05). The most frequent genotypes were *HOTAIR* rs10783618*T/T (38%), *LINC-ROR* rs1942347*A/A (40%), and *MALAT1* rs3200401*C/T (47%) (Figure 1). MAF of *HOTAIR* rs10783618*C, *LINC-ROR* rs1942347*T, and *MALAT1* rs3200401*T were 0.45, 0.42, and 0.36, respectively, in our Caucasian population. The overall MAF of ancestral alleles in the 1000 Genome project (www.ensembl.org; last accessed on 20 August 2021) were 0.50 (rs10783618*C allele), 0.47 (rs1942347*T allele), and 0.14 (rs3200401*T allele). Genotyping of the two study populations revealed higher frequency of *HOTAIR* rs10783618*C/C (35% vs. 23%, *p* = 0.05) and *MALAT1* rs3200401*T/T (18% vs. 7%, *p* < 0.001) in carrier SLE patients compared to controls (Table 2).

### 3.3. Association of lncRNA Variants with SLE Development

**Figure 2:**
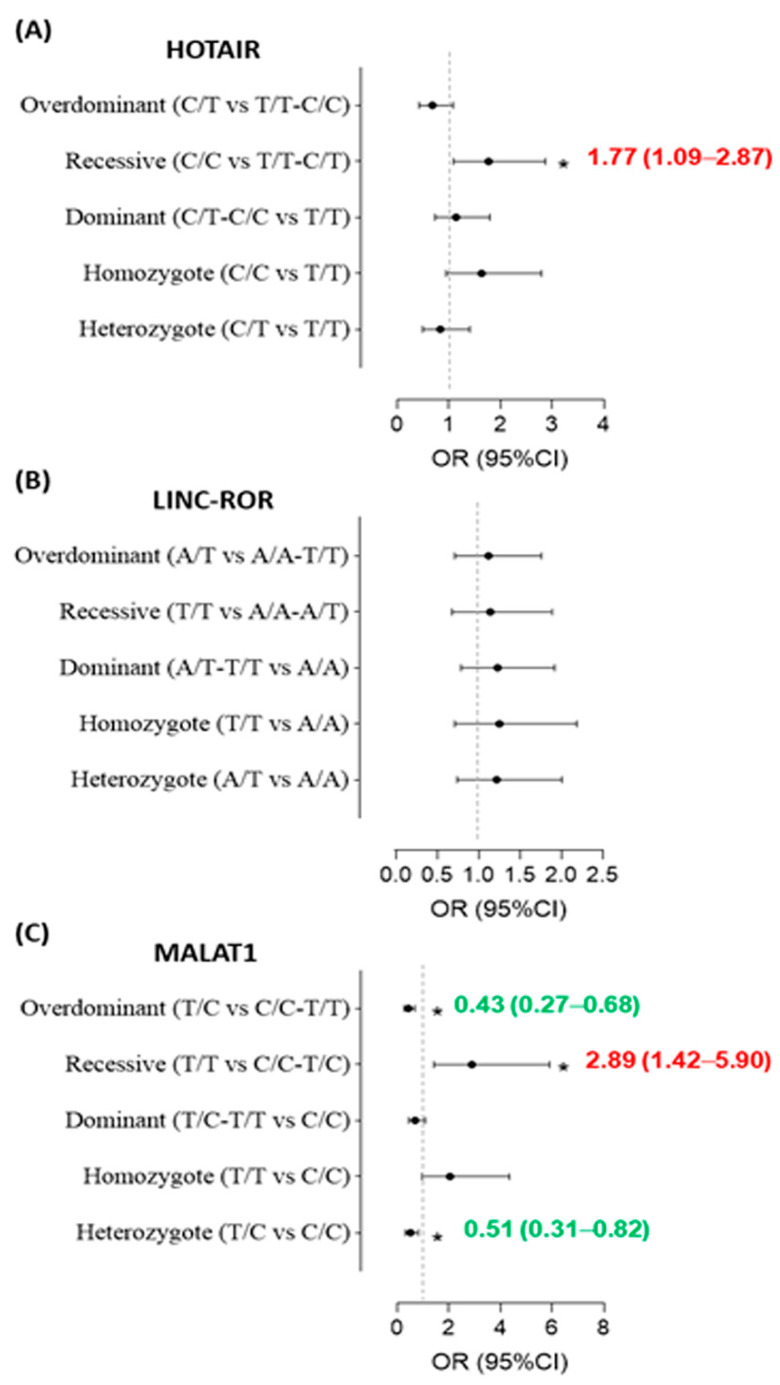
Risk of systemic lupus erythematosus by genetic association models of *HOTAIR* (**A**), *LINC-ROR* (**B**), and *MALAT1* (**C**)_genotypes. A Chi-square test was used. OR (95%CI), odds ratio, and confidence interval. * Statistically significant *p*-value ≤ 0.05. Adjusted covariates: age and sex. Increased/decreased susceptibility labeled with (red/green OR 95%CI), respectively.

As depicted in Figure 2 in the genetic association models, after adjustment by age and sex, *HOTAIR* rs10783618*C/C was associated with 77% increased risk of SLE (OR = 1.77, 95%CI = 1.09–2.87, *p* = 0.020) under the recessive model. Similarly, *MALAT1* rs3200401*T/T carriers were three times more likely to develop SLE (OR = 2.89, 95%CI = 1.42–5.90) under the recessive model. In contrast, carrying a single C allele of *MALAT1* gene conferred protection. Heterozygotes of *MALAT1* rs3200401*T/C were associated with a 49–57% decreased risk of SLE under codominant (OR = 0.51, 95%CI = 0.31–0.82, *p* < 0.001) and over-dominant models (OR = 0.43, 95%CI = 0.27–0.68, *p* < 0.001). 

### 3.4. Association of lncRNA Variants with Clinic-Laboratory Variables

*LINC-ROR* rs1942347*A/A patients were more likely to have a positive family history of SLE, whereas *HOTAIR* rs10783618*C/C was associated with higher frequency of arthritis (*p* = 0.001) and the presence of oral ulcers (*p* = 0.002), while patients carrying *HOTAIR* rs10783618*T/T genotype were more likely to develop hair loss (*p* < 0.001), weight loss (*p* = 0.001), and neurological symptoms (*p* = 0.003). Despite being associated with higher disease risk, *MALAT1* rs3200401*T/T exhibited the least frequency of neurological features (*p* = 0.001) (Table 3). 

### 3.5. Impact of lncRNA Variants on the Disease Activity Index

**Figure 3:**
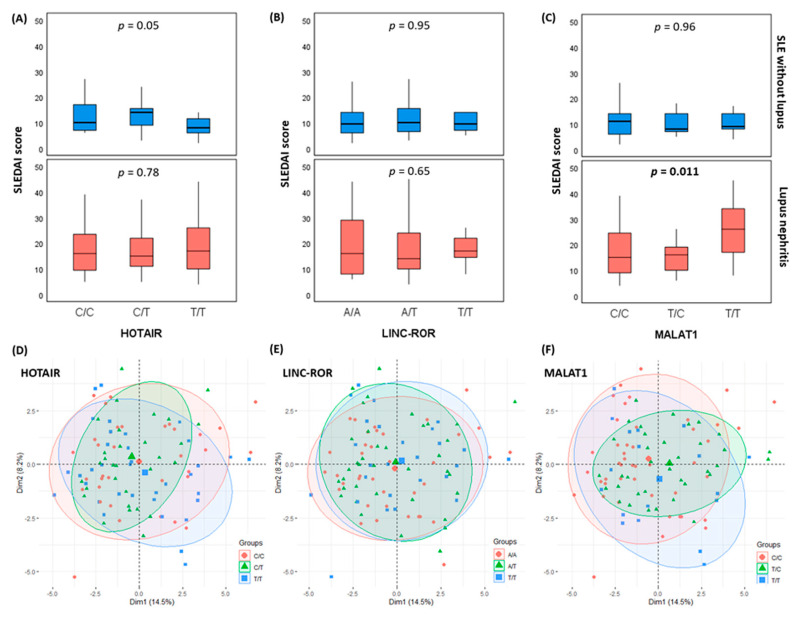
Impact of variant on disease activity index. (**A**–**C**) Box plots in SLE with and without nephritis show no significant difference in SLE disease activity index (SLEDAI). (**D**–**F**) The principal component analysis for data exploration showed no clear demarcation between patients with different genotypes. *p*-values ≤ 0.05 were considered statistically significant.

The principal component analysis for data exploration showed no clear demarcation between SLE patients carrying different genotypes regarding the disease activity index (Figure 3). 

## 4. Discussion

Growing evidence has unleashed the critical regulatory role of lncRNAs in autoimmune and inflammatory conditions [32,33]. Additionally, lncRNA and other genetic/epigenetic factors such as circulating tumor DNA (ctDNA) and microRNAs (miRNAs) have been investigated as biomarkers to support diagnosis, prognosis, and the prediction of treatment response in cancer and several autoimmune disorders. Unlike ctDNA, ncRNAs (miRNAs and lncRNAs) are very stable since they are primarily released in vesicles or associated with other proteins [34,35,36]. Thus, lncRNA may represent a robust tool for studying molecular heterogeneity and clonal divergence in diseases, which might be of significant importance, especially in the era of personalized medicine. For example, several lncRNAs have been dysregulated in melanoma, including HOTAIR, BANCR, UCA1, and MALAT-1, and related to invasion and metastasis. Furthermore, it was found that MALAT1 knockdown was followed by a decrease in melanoma cell migration, whereas HOTAIR knockdown was associated with suppression of cell motility and invasive potential [37,38,39], confirming the clinical utility of the studied lncRNAs.

Abnormal expression and function of lncRNAs are tightly linked to the pathogenesis of SLE [21,22,40]. However, knowledge about the impact of genetic variants of these lncRNAs remains limited, and only a few polymorphisms within lncRNA genes were reported. Two examples of the A > G mutation at rs13259960 in SLEAR and the risk variants rs205764 and rs547311 in the promoter region of linc00513 SLE-related lncRNA genes have shown an association with susceptibility to SLE [19,41]. In this study, we aimed to explore the contribution of the *HOTAIR*, *LINC-ROR*, and *MALAT1* polymorphisms to the susceptibility of developing SLE. To the best of our knowledge, this is the first report to spotlight the role of these polymorphisms in contributing to SLE disease in humans. In this study, the genotyping of blood samples from SLE patients and healthy donors revealed that the homozygosity of the mutant alleles of *HOTAIR* and *MALAT1* genes was associated with higher disease risk. 

The role and function of HOTAIR have not yet been annotated in the exact etiology of SLE. In the current analysis, we elucidated the putative role of the HOTAIR genetic variant in the pathogenesis of SLE by investigating the genotypes associated with disease risk in Caucasian SLE patients and healthy controls. We found that the *HOTAIR* rs10783618*C/C was associated with a 77% increased risk of SLE compared to T/T and C/T. In other disorders, the maternal and placental *HOTAIR* rs10783618 polymorphism conferred increased preeclampsia susceptibility [42]. The same SNP was studied in Chinese gastric cancer patients but did not significantly differ between cancer and noncancer blood samples [43,44]. The *HOTAIR* rs10783618 SNP is positioned in a well-conserved region across multiple mammalian species. Studies showed it had no impact on the HOTAIR mRNA splicing or secondary structure of mRNA. It was suggested that the SNP might create or alter exonic splicing silencers and/or exonic splicing enhancers [42]. HOTAIR lncRNA can play a major role in epigenetic regulation by modifying chromatin structure [45]. It can modulate a series of genes related to immune and inflammatory disorders. It promotes arthritis progression via miR-17-5p/FUT2/β-catenin axis [46], and cartilage degradation in osteoarthritis by inhibiting WIF-1 expression and activating the Wnt pathway [47]. HOTAIR modulates chondrocyte apoptosis and inflammation in osteoarthritis via the regulation of the miR-1277-5p/SGTB axis [48]. HOTAIR induces GLI2 expression through Notch signaling in systemic sclerosis dermal fibroblasts [49]. HOTAIR/miR-34a-5p/Notch1 signaling pathway may regulate the development of intervertebral disc degeneration [45]. HOTAIR promotes renal interstitial fibrosis via the modulation of miR-124 expression and regulation of the NOTCH1 signaling pathway [50]. Blocking HOTAIR protects human chondrocytes against IL-1beta-induced cell apoptosis, ECM degradation, inflammatory response, and oxidative stress via regulating miR-222-3p/ADAM10 axis [51]. HOTAIR knockdown alleviates gouty arthritis through miR-20b upregulation and NLRP3 downregulation [52]. SLE is a complex autoimmune disease with obscure etiology. Our findings showed the HOTAIR variant conferred a predisposition to SLE. Collectively, these results might offer a piece of the puzzle in the etiology of SLE. Further functional studies combined with animal research are warranted to enhance our understanding of the molecular interaction associated with HOTAIR gene variants.

Increasing reports have indicated that MALAT1 plays a critical role in inflammation and immunological diseases. It is aberrantly expressed in diverse inflammatory diseases and exerts a proinflammatory effect by increasing the levels of multiple cytokines [53]. It has been regarded as a key regulator of the NF-κB signaling related to inflammation [54]. MALAT1 is upregulated in osteoarthritis and facilitates cartilage ECM degradation in IL-1β-induced chondrocytes [55]. MALAT1 enhances the levels of proinflammatory cytokines (IL-18 and IL-1β) in pregnancy-induced hypertension by activating the NF-κB pathway [56]. Its suppression reduced proinflammatory cytokines production by regulating miR-150-5p/ZBTB4 axis via JAK/STAT signal pathway in systemic juvenile idiopathic arthritis [57]. In the current study, *MALAT1* rs3200401*T/T was associated with three times more risk than C/C and C/T under the recessive inheritance model.

In contrast, heterozygosity of *MALAT1* rs3200401*T/C was associated with a 49–57% decreased risk of SLE. MALAT1 is an abundantly expressed lncRNA localized to nuclear speckles and has been associated with gene expression regulation [58]. MALAT-1 expression was overexpressed in primary mononuclear cells of SLE patients and predominantly in primary monocytes [26]. In vitro studies showed MALAT-1 as a critical regulatory factor in the pathogenesis of SLE. MALAT1 exerts its detrimental effects by regulating the SIRT1 signaling pathway. Knockdown of MALAT1 in both THP-1 cell lines and human primary monocytes by small interfering RNA (siRNA) significantly reduced the expression of IL-21, a well-known inflammatory cytokine secreted from monocytes [26]. The rs3200401 SNP has not been studied before in SLE. However, McCown et al. demonstrated the location of the SNP in the binding site of miR-217-5p. RNAfold software predicts that the SNP may decrease the stability of the hairpin domain and reduce the number of unpaired nucleotides in the internal loops, yielding the binding site less accessible to the microRNA [59]. Such single point alteration at the sequence level can perturb the secondary structure of MALAT1 or modify its dynamic interacting partners, leading to profound biological consequences. Our findings as to it being a risky gene highlight its putative role as a diagnostic genetic biomarker for SLE. Further association studies in diverse ethnic groups and functional studies are necessary to confirm our findings.

Though our study, to the authors’ knowledge, is the first to uncover the significant association of the studied variants with SLE susceptibility, it had some limitations. First, it has a relatively limited sample size due to limited time and funds. Second, as all participants were included from the same population, the generalizability of the findings is limited.

## 5. Conclusions

Our study provides new insights into the genetics of SLE and extends the role of lncRNAs in the pathogenesis of SLE. The lncRNAs, *HOTAIR*, and *MALAT1* gene polymorphisms confer susceptibility for SLE, providing a potential theoretical foundation for their clinical translation in SLE disease. Further independent studies with different races and larger sample sizes are necessary to elucidate the molecular mechanisms underlying these findings.

## Figures and Tables

**Table 1:** Characteristics of SLE patients with/without lupus nephritis.

Characteristics		Total (N = 163)	SLE without Nephritis (N = 65)	Lupus Nephritis (N = 98)	*p*-Value
**Demographics**					
Age, years	Mean ± SD	35.81 ± 9.60	34.86 ± 9.59	36.45 ± 9.61	0.38
Sex	Male	16 (9.8)	11 (16.9)	5 (5.1)	**0.016**
	Female	147 (90.2)	54 (83.1)	93 (94.9)	
Family history	Negative	105 (64.4)	40 (61.5)	65 (66.3)	0.61
	Positive	58 (35.6)	25 (38.5)	33 (33.7)	
**Clinical manifestations**					
Organ involvement	Malar rash	109 (66.9)	45 (69.2)	64 (65.3)	0.61
	Discoid rash	77 (47.2)	26 (40)	51 (52)	0.15
	Photosensitivity	61 (37.4)	23 (35.4)	38 (38.8)	0.74
	Hair loss	129 (79.1)	52 (80)	77 (78.6)	0.82
	Oral ulcer	46 (28.2)	19 (29.2)	27 (27.6)	0.86
	Arthritis	81 (49.7)	31 (47.7)	50 (51)	0.75
	Ecchymosis	19 (11.7)	8 (12.3)	11 (11.2)	0.83
	Fever	30 (18.4)	12 (18.5)	18 (18.4)	0.98
	Infection	26 (16)	9 (13.8)	17 (17.3)	0.55
	Dyspnea	68 (41.7)	30 (46.2)	38 (38.8)	0.42
	Chest pain	35 (21.5)	11 (16.9)	24 (24.5)	0.33
	Cough	35 (21.5)	14 (21.5)	21 (21.4)	0.98
	CNS	41 (25.2)	18 (27.7)	23 (23.5)	0.54
	Peripheral neuropathy	70 (42.9)	28 (43.1)	42 (42.9)	0.98
	Hematuria	56 (34.4)	20 (30.8)	36 (36.7)	0.43
	Weight loss	76 (46.6)	36 (55.4)	40 (40.8)	0.07
**Severity**					
SLEDAI score	Mean ± SD	15.97 ± 9.82	12.03 ± 7.75	18.63 ± 10.21	**<0.001**
	Grade 1	12 (7.4)	9 (13.8)	3 (3.1)	**<0.001**
	Grade 2	50 (30.7)	26 (40)	24 (24.5)	
	Grade 3	56 (34.4)	22 (33.8)	34 (34.7)	
	Grade 4	45 (27.6)	8 (12.3)	37 (37.8)	
Markers for severity	Elevated inflammatory markers	67 (41.1)	30 (46.2)	37 (37.8)	0.33
	Thrombocytopenia	5 (3.1)	1 (1.5)	4 (4.1)	0.64
	Hypocomplementemia	47 (28.8)	16 (24.6)	31 (31.6)	0.38
	High serum creatinine	72 (44.2)	16 (24.6)	56 (57.1)	**<0.001**
	Proteinuria	92 (56.4)	15 (23.1)	77 (78.6)	**<0.001**
	Cast in the urine	29 (17.8)	4 (6.2)	25 (25.5)	**0.001**
**Laboratory data**					
Autoantibodies	Positive dsDNA	147 (90.2)	51 (78.5)	96 (98)	**<0.001**
	Positive ANA titer	162 (99.4)	64 (98.5)	98 (100)	0.21
Biochemical tests	Hemoglobin (g/dL)	11.66 ± 2.89	11.77 ± 1.50	11.58 ± 3.54	0.69
	RBC (×10^6^ per mm^3^)	4.09 ± 0.74	4.16 ± 0.72	4.05 ± 0.75	0.42
	HCT (%)	38.18 ± 6.05	38.53 ± 6.38	37.95 ± 5.85	0.42
	MCV (fl)	81.42 ± 6.36	81.87 ± 6.72	81.11 ± 6.12	0.42
	Platelet count (×10^3^/mm^3^)	264.51 ± 77.59	256.48 ± 80.57	269.92 ± 75.46	0.37
	WBC (×10^3^/uL)	6.58 ± 2.22	6.64 ± 2.21	6.55 ± 2.24	0.67
	Neutrophil (%)	63.30 ± 10.46	62.44 ± 11.27	63.88 ± 9.89	0.40
	Lymphocyte (%)	30.01 ± 9.66	30.91 ± 9.95	29.41 ± 9.46	0.62
	C3 (mg/dL)	95.52 ± 47.86	96.46 ± 47.69	94.89 ± 48.21	0.90
	C4 (mg/dL)	27.94 ± 15.62	27.78 ± 15.78	28.04 ± 15.60	0.91
	CRP (mg/L)	2.95 ± 2.89	3.04 ± 3.22	2.88 ± 2.67	0.69
	ESR 1st hour	26.84 ± 13.58	27.67 ± 14.91	26.28 ± 12.65	0.93
	ALT (U/L)	26.61 ± 9.62	27.00 ± 8.32	26.35 ± 10.44	0.81
	AST (U/L)	26.50 ± 8.48	27.02 ± 8.49	26.16 ± 8.50	0.76
	Serum creatinine (mg/dL)	1.18 ± 1.19	0.99 ± 0.28	1.31 ± 1.51	0.11
	Blood urea (mg/dL)	35.11 ± 11.86	32.63 ± 6.96	36.78 ± 14.04	**0.05**

Values are shown as number (%) or mean ± standard deviation (SD). Chi-square and Student’s *t*-tests were used. Bold values are considered statistically significant at *p*-value < 0.05. CNS: central nervous system; SLEDAI: systemic lupus erythematosus disease activity index; dsDNA: double-stranded deoxyribonucleic acid; ANA: antinuclear antibody; RBC: red blood cell; HCT: hematocrit; MCV: mean cell volume; WBC: white blood cell; C3/4, complement 3/4; CRP: C-reactive protein; ALT: alanine transaminase; AST: aspartate transaminase.

**Table 2:** Genotype and allele frequencies of *HOTAIR*, *LINC-ROR*, and *MALAT1* polymorphisms.

Variable	Controls		Cases		*p*-Value
	Count	Proportion	Count	Proportion	
*HOTAIR* (rs10783618)					
Allele					
T	190	0.58	166	0.51	0.059
C	136	0.42	160	0.49	
Genotypes					
C/C	38	0.23	57	0.35	**0.05**
T/C	60	0.37	46	0.28	
T/T	65	0.4	60	0.37	
*LINC-ROR* (rs1942347)					
Allele					
A	194	0.6	182	0.56	0.34
T	132	0.4	144	0.44	
Genotypes					
A/A	69	0.42	61	0.37	0.66
A/T	56	0.34	60	0.37	
T/T	38	0.23	42	0.26	
*MALAT1* (rs3200401)					
Allele					
C	210	0.64	206	0.63	0.74
T	116	0.36	120	0.37	
Genotypes					
C/C	59	0.36	73	0.45	**<0.001**
C/T	92	0.56	60	0.37	
T/T	12	0.07	30	0.18	

Values are shown as numbers (%). A Chi-square test was used. Bold *p*-value ≤ 0.05 was considered statistically significant.

**Table 3:** Association of lncRNA polymorphisms with clinical parameters.

Variables	*HOTAIR*			*p*-Value	*LINC-ROR*			*p*-Value	*MALAT1*			*p*-Value
	C/C	T/C	T/T		A/A	A/T	T/T		C/C	C/T	T/T	
	95	106	125		130	116	80		132	152	42	
Early onset	68.4%	80.4%	60.0%	0.08	68.9%	66.7%	71.4%	0.88	60.0%	70.0%	71.2%	0.52
Female gender	89.5%	89.1%	91.7%	0.89	93.4%	91.7%	83.3%	0.21	96.7%	90.0%	87.7%	0.38
Positive FH	36.8%	47.8%	25.0%	0.05	47.5%	31.7%	23.8%	**0.034**	36.7%	26.7%	42.5%	0.16
Malar rash	63.2%	54.3%	80.0%	**0.016**	62.3%	75.0%	61.9%	0.24	66.7%	71.7%	63.0%	0.57
Discoid rash	42.1%	45.7%	53.3%	0.46	42.6%	51.7%	47.6%	0.61	46.7%	58.3%	38.4%	0.07
Photosensitivity	36.8%	45.7%	31.7%	0.33	39.3%	33.3%	40.5%	0.71	26.7%	38.3%	41.1%	0.38
Hair loss	57.9%	84.8%	95.0%	**<0.001**	78.7%	81.7%	76.2%	0.79	86.7%	85.0%	71.2%	0.08
Oral ulcers	36.8%	39.1%	11.7%	**0.002**	27.9%	28.3%	28.6%	1.00	23.3%	28.3%	30.1%	0.78
Arthritis	68.4%	45.7%	35.0%	**0.001**	49.2%	40.0%	64.3%	0.05	43.3%	56.7%	46.6%	0.38
Fever	26.3%	13.0%	15.0%	0.16	18.0%	20.0%	16.7%	0.91	23.3%	26.7%	9.6%	**0.030**
Recurrent infection	21.1%	13.0%	13.3%	0.43	19.7%	13.3%	14.3%	0.60	13.3%	13.3%	19.2%	0.60
Weight loss	26.3%	58.7%	56.7%	**0.001**	45.9%	53.3%	38.1%	0.31	50.0%	48.3%	43.8%	0.80
Ecchymosis	15.8%	13.0%	6.7%	0.29	13.1%	8.3%	14.3%	0.59	6.7%	18.3%	8.2%	0.12
Neurological	19.3%	13.0%	40.0%	**0.003**	23.0%	28.3%	23.8%	0.77	20.0%	41.7%	13.7%	**0.001**
Hematuria	26.3%	26.1%	48.3%	**0.02**	31.1%	40.0%	31.0%	0.51	30.0%	43.3%	28.8%	0.18
Lupus nephritis	61.4%	56.5%	61.7%	0.84	54.1%	61.7%	66.7%	0.42	56.7%	61.7%	60.3%	0.90
Pulmonary	54.4%	54.3%	53.3%	0.99	50.8%	58.3%	52.4%	0.69	56.7%	60.0%	47.9%	0.36

Data are presented as a percentage. FH: family history. A two-sided Chi-square test was used. Bold *p*-values ≤ 0.05 were considered statistically significant.

## Data Availability

All generated data in this study are included in the article.
